# Population‐based study evaluating and predicting the probability of death resulting from thyroid cancer among patients with papillary thyroid microcarcinoma

**DOI:** 10.1002/cam4.2597

**Published:** 2019-10-06

**Authors:** Kun Wang, Jing Xu, Shuyu Li, Shiyang Liu, Lin Zhang

**Affiliations:** ^1^ Department of Thyroid and Breast Surgery Tongji Hospital of Tongji Medical College of Huazhong University of Science and Technology Wuhan P.R. China

**Keywords:** competing risk, nomogram, papillary thyroid microcarcinoma, SEER

## Abstract

**Background:**

Papillary thyroid microcarcinoma (PTMC) is an indolent carcinoma. The cumulative incidence of death from patients with PTMC and the nomogram built based on the competing risks model have not been described.

**Methods:**

Patients diagnosed with PTMC were selected from the Surveillance, Epidemiology, and End Results (SEER) program (1983‐2015). Cumulative incidence function was utilized to calculate the likelihood of death resulting from thyroid cancer. Gray's test was conducted to examine the difference in the cumulative incidence of death between groups. A proportional subdistribution hazard model was constructed and we further built a nomogram to quantify the likelihood of death using the model. A 10‐fold cross‐validation procedure was adopted to validate the model.

**Results:**

A total of 46 662 patients diagnosed with PTMC were included. The median follow‐up time was 81 months (range, 1 to 407 months). The 5‐year, 10‐year, and 20‐year probabilities of death from thyroid carcinoma were 0.3%, 0.6%, and 1.4%, respectively. The age at diagnosis, sex, tumor extension, and lymph node involvement were related to the cumulative incidence of death. A proportional subdistribution hazard model was developed. The performance of the model was good. A nomogram was built based on the model to predict the likelihood of death in patients with PTMC.

**Conclusion:**

The survival rate of patients with PTMC is excellent. The nomogram constructed based on the well‐performed competing risks model is helpful for both patients and clinicians.

## Graphical abstract

The probability of death from papillary thyroid microcarcinoma is very low. The nomogram constructed based on the competing risks model is helpful to predict the cumulative incidence of death from papillary thyroid microcarcinoma.

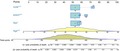

## 1 INTRODUCTION

The incidence rate of thyroid cancer has been rising rapidly all over the world in recent decades.^1^, ^2^ Papillary thyroid carcinoma accounts for about 90% of thyroid carcinoma. Papillary thyroid carcinoma measuring less than or equal to 1 cm in the maximum diameter is called papillary thyroid microcarcinoma (PTMC),^3^ which accounts for half of the newly diagnosed papillary thyroid carcinoma. Although the morbidity rate of thyroid cancer has risen, the mortality from the cancer has been stable.^4^ PTMC tends to have an indolent nature and can remain asymptomatic for long periods of time. Two Japanese studies of PTMC showed similar outcomes of active surveillance compared with surgical treatment.^5^, ^6^ Nevertheless, there are no reliable clinical features to differentiate the minority of PTMC patients who will develop progression from the patients with indolent PTMC that will not result in clinically significant disease. Thus, the active surveillance approach is rarely adopted beyond the former two centers.

The disease‐specific mortality rate of PTMC has been reported less than 1%.^7^ An autopsy study indicated that almost 9% of cadavers had thyroid cancer, even though the cause of death was nonthyroidal disease.^8^ Hence, competing risks can be a crucial problem when researchers conduct a survival analysis in which the event of interest is death resulting from thyroid carcinoma. Competing risks will arise in the survival analysis when the event of interest is impeded by a prior event of a different type,^9^ such as death from thyroid cancer and death from other causes. The occurrence of one of the two events will prevent another event from ever happening. The standard analysis of time‐to‐event data such as the Kaplan‐Meier estimate may no longer be applicable if competing risks occur, because censoring of the competing event in the survival analysis will result in informative censoring and causes bias. Instead of being censored, subjects who experience the competing event are still at the risk of experiencing the event of interest in the proportional subdistribution hazard model. Thus, the proportional subdistribution hazard model can be applied to predict the cumulative incidence for an event of interest based on covariates.^10^

In this study, we calculated the cumulative incidence of death resulting from PTMC using the data from Surveillance, Epidemiology, and End Results (SEER) program, and provided clinicians with a practical nomogram which was built based on a proportional subdistribution hazard model to predict the likelihood of death in patients with PTMC.

## 2 PATIENTS AND METHODS

### 2.1 Selection of patients

SEER program of the National Cancer Institute is the largest population‐based cancer database in the US which covers approximately 34.6% of the population.^11^ The study population was obtained from the SEER program. The data from SEER program contain unrecognizable patient information and are publicly accessible for cancer‐based study. The approval of the institutional review boards and the patient's informed consent were not required.

**Figure 1:**
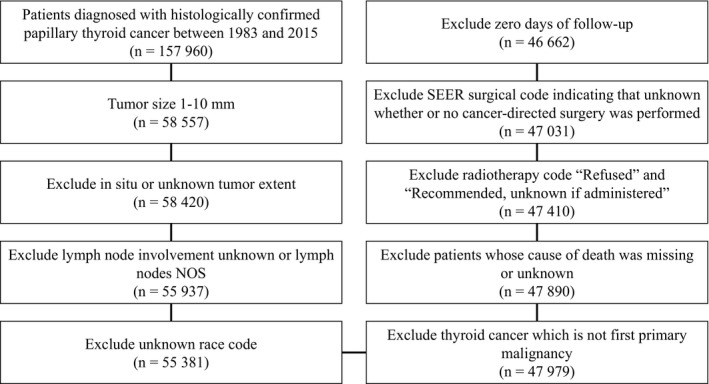
Flow chart for selection of the SEER data set

Using SeerStat 8.3.4, patients who were diagnosed with papillary thyroid carcinoma between 1983 and 2015 were identified and extracted for the study.^12^ The diagnosis of papillary thyroid cancer was determined by a primary site code of 739 in combination with the International Classification of Diseases for Oncology, third edition (ICD‐O‐3) codes: 8050/3, 8260/3, 8340/3, 8341/3, 8342/3, 8343/3, and 8344/3. Only histologically confirmed thyroid cancer with size from 1 to 10 mm was selected. We excluded thyroid carcinoma which was diagnosed only on autopsy or by death certificate. Other exclusion criteria and the flow chart for data selection are shown in Figure 1. Finally, there were 46 662 patients with PTMC included in the study cohort.

### 2.2 Assessment of variables

Clinicopathological variables such as age at diagnosis, sex, race, tumor size, histologic subtype, tumor extent, lymph node involvement, and radiotherapy were obtained from the database. Age at diagnosis was categorized as less than 45, 45‐64, and more than or equal to 65 years and tumor size was categorized as less than 6 and 6 to 10 millimeters to compare the cumulative incidence of death among groups. The histologic subtype was determined by ICD‐O‐3 and grouped as 8050/3: Papillary carcinoma, NOS; 8260/3: Papillary adenocarcinoma, NOS; 8341/3: Papillary microcarcinoma; 8340/3: Papillary carcinoma, follicular variant; 8342/3: Papillary carcinoma, oxyphilic cell; 8343/3: Papillary carcinoma, encapsulated; and 8344/3: Papillary carcinoma, columnar cell. Tumor extent was documented as localized (confined to the thyroid gland), capsular extension (extension into thyroid capsule, but not beyond), or further extension (extension into adjacent tissue or metastasis).

### 2.3 Statistical analysis

The likelihood of death was calculated by the cumulative incidence function with Gray's test.^13^ The competing risks model was constructed using Fine and Gray proportional subdistribution hazards model.^14^ To avoid overfitting, Bayesian information criteria were selected to perform the model selection. For model validation, a 10‐fold cross‐validation procedure was adopted to assess both calibration and discrimination.^15^ The calibration plot was utilized to examine whether the predicted probability of death from our nomogram matched with the observed probability of death at a certain time point. The area under operating characteristic curve (AUC) was used to evaluate the discrimination of a model. Furthermore, we plotted AUC across all times to evaluate the predictive accuracy of the model during the whole follow‐up time. All statistical analyses were performed using R version 3.6.0 software with package cmprsk and riskRegression. Two‐sided *P* < .05 was considered statistically significant.

## 3 RESULTS

### 3.1 Patient characteristics

Our study involved 46 662 patients with histologically confirmed PTMC. The clinicopathological characteristics of the study cohort are displayed in Table 1. Overall, most patients were females (82.2%), were White (84.0%), were diagnosed as papillary adenocarcinoma (8260/3, 49.0%), and at a local stage (85.1%). The median age at diagnosis was 49 years (range, 3 to 96 years). Median follow‐up time was 81 months (range, 1 to 407 months). There were 2764 death events (5.9%), including 270 deaths (0.6%) from thyroid cancer and 2494 (5.3%) deaths from other causes.

**Table 1:** Patient demographics and clinical characteristics

Demographic or characteristic	All patients (n = 46 662)	
	No.	%
Age at diagnosis, y		
Mean (SD)	49.2 (13.8)	
Median [Min, Max]	49.0 [3.0, 96.0]	
Sex		
Female	38 377	82.2
Male	8285	17.8
Race		
Black	2942	6.3
White	39 195	84.0
Other	4525	9.7
Tumor size, mm		
Mean (SD)	5.4 (3.0)	
Median [Min, Max]	5.0 [1.0, 10.0]	
Histologic subtype		
8050/3: Papillary carcinoma, NOS	5252	11.3
8260/3: Papillary adenocarcinoma, NOS	22 842	49.0
8341/3: Papillary microcarcinoma	4506	9.7
8340/3: Papillary carcinoma, FV	13 551	29.0
8342/3: Papillary carcinoma, oxyphilic cell	48	0.1
8343/3: Papillary carcinoma, encapsulated	232	0.5
8344/3: Papillary carcinoma, columnar cell	231	0.5
Extent of tumor		
Localized	39 712	85.1
Capsular extension	1667	3.6
Further extension	5283	11.3
Lymph node involvement		
No involvement	40 764	87.4
Lymph node involvement	5898	12.6
Radiotherapy		
None	33 206	71.2
Yes	13 456	28.8
Death resulting from thyroid cancer	270	0.6
Death resulting from other causes	2494	5.3
Follow‐up, mo		
Mean (SD)	96.0 (70.0)	
Median [Min, Max]	81.0 [1.00, 407]	

Abbreviations: FV, follicular variant; NOS, not otherwise specified; SD, standard deviation.

### 3.2 Probability of death

**Figure 2:**
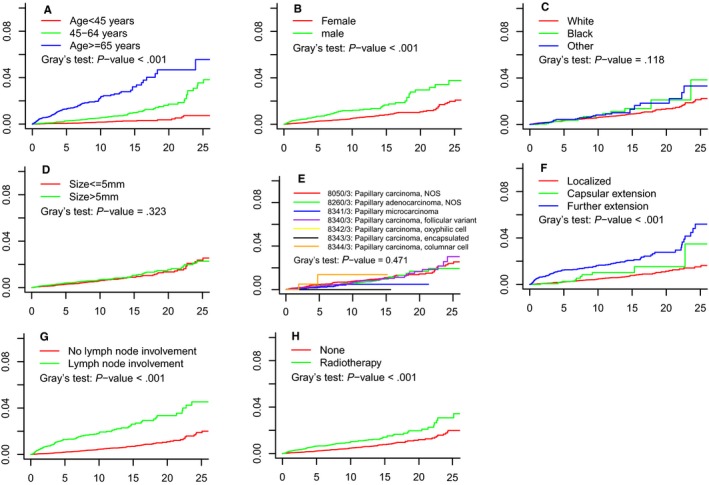
Cumulative incidence estimates of death by (A) age, (B) sex, (C) race, (D) size, (E) histology subtype, (F) extent of tumor, (G) lymph node involvement, and (H) radiotherapy. The x‐axis designates time since diagnosis (y). The y‐axis indicates probability of death resulting from thyroid cancer (%)

The cumulative incidence of death for all patients resulting from PTMC and other causes based on different clinicopathological characteristics is shown in Table 2. The 5‐year, 10‐year, and 20‐year cumulative incidences of death resulting from PTMC were 0.3%, 0.6%, and 1.4%, respectively. As for death resulting from other causes, the cumulative incidences were 2.3%, 6.0%, and 10.6%, respectively. Gray's test indicated that the cumulative incidence of death resulting from PTMC increased with age (*P* < .001). Furthermore, extent of tumor, sex, lymph node involvement, and radiotherapy also showed significant associations with the cumulative incidence of death (*P* < .001). However, race, tumor size, and histologic subtype were not significantly associated with the cumulative incidence of death. Cumulative incidence of death resulting from PTMC during the whole follow‐up time is presented in Figure 2.

**Table 2:** 5‐, 10‐, and 20‐year cumulative incidences of death among patients with thyroid cancer

Characteristic	Cumulative incidence of death resulting from thyroid cancer				Cumulative incidence of death resulting from other causes			
	5 y (%)	10 y (%)	20 y (%)	*P* a	5 y (%)	10 y (%)	20 y (%)	*P* a
All patients	0.3	0.6	1.4		2.3	6.0	15.9	
Age at diagnosis, y				<.001				<.001
≤44	0.1	0.2	0.4		0.6	1.4	4.1	
45‐64	0.3	0.5	1.7		2.0	5.4	16.6	
≥75	1.3	2.3	4.7		7.9	21.9	61.4	
Sex				<.001				<.001
Female	0.3	0.5	1.1		2.0	5.2	14.2	
Male	0.7	1.2	3.0		4.0	9.8	23.1	
Race				.12				<.001
Black	0.3	0.8	2.1		4.0	10.4	25.5	
White	0.3	0.6	1.4		2.3	6.0	15.6	
Other	0.4	0.8	1.8		1.5	3.6	13.2	
Tumor size, mm				.32				<.001
≤5	0.3	0.6	1.3		2.7	6.8	17.9	
6‐10	0.4	0.7	1.5		1.9	5.1	13.6	
Histologic subtype				.47				<.001
8050/3: Papillary carcinoma, NOS	0.5	0.8	1.4		2.3	5.8	15.0	
8260/3: Papillary adenocarcinoma, NOS	0.3	0.6	1.7		2.0	5.4	14.9	
8341/3: Papillary microcarcinoma	0.2	0.5	0.5		2.4	6.8	14.2	
8340/3: Papillary carcinoma, FV	0.3	0.7	1.7		2.8	6.8	18.3	
8342/3: Papillary carcinoma, oxyphilic cell	0	0	—		0	5.9	—	
8343/3: Papillary carcinoma, encapsulated	0	0	—		2.4	5.9	—	
8344/3: Papillary carcinoma, columnar cell	1.4	1.4	—		0	4.3	—	
Extent of tumor				<.001				<.001
Localized	0.2	0.5	1.1		2.4	6.2	16.7	
Capsular extension	0.3	1.0	1.5		1.6	4.5	16.3	
Further extension	1.2	1.6	2.8		2.2	5.4	13.6	
Lymph node involvement				<.001				<.001
No involvement	0.2	0.5	0.1		2.3	6.2	16.7	
Lymph node involvement	1.3	1.9	0.3		2.3	5.0	12.4	
Radiotherapy				<.001				<.001
None	0.2	0.5	1.2		2.6	6.8	17.5	
Yes	0.6	1.0	2.0		1.6	4.4	12.4	

Abbreviations: FV, follicular variant; NOS, not otherwise specified.

a Gray's test.

### 3.3 Nomogram

**Figure 3:**
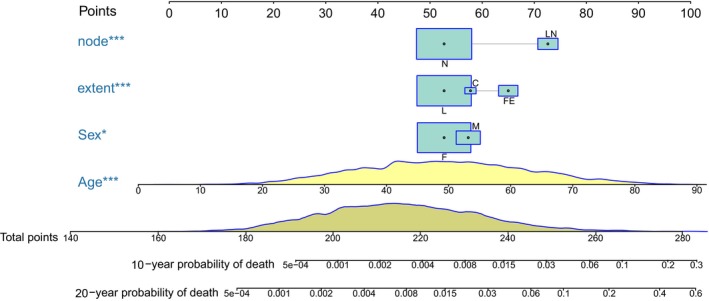
Prognostic nomogram for predicting 10‐ and 20‐year probability of death resulting from papillary thyroid microcarcinoma. N, no lymph node involvement; LN, lymph node involvement; L, localized; C, capsular extension; FE, further extension; F, female; M, male. *P < .05; **P < .01; ***P < .001

**Figure 4:**
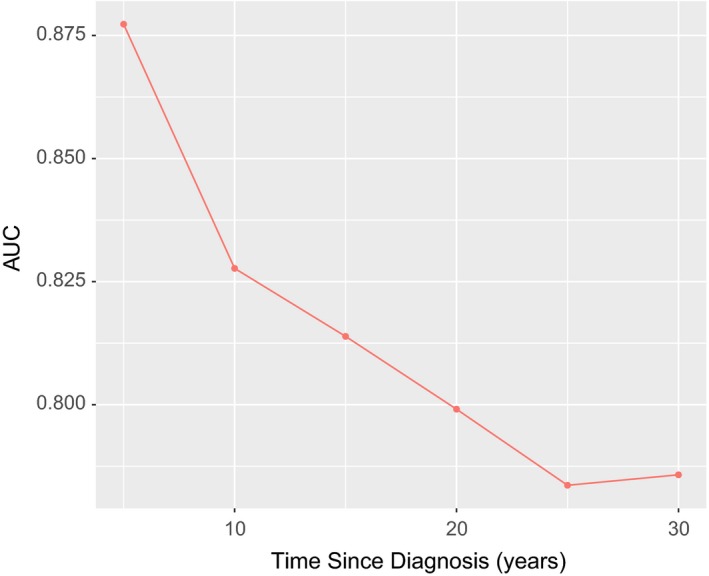
The area under operating characteristic curve (AUC) over follow‐up time

**Figure 5:**
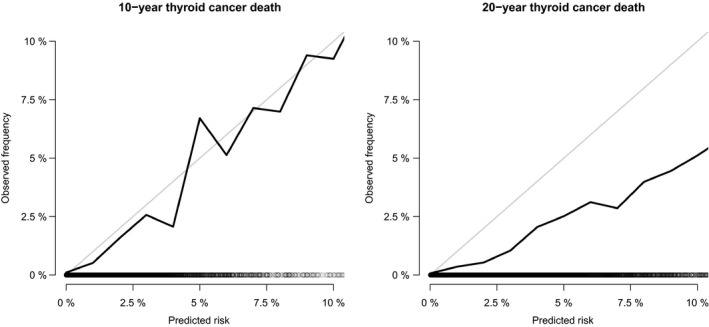
Calibration curves obtained by using cross‐validation method

The proportional subdistribution hazard model comprising all the variables showed the effect of clinicopathological characteristics on the cumulative incidence of death resulting from PTMC (Table 3). Age, extent of tumor, and lymph node involvement showed significant statistical contributions to the model (*P* < .001). Male, race, and radiotherapy showed only marginally statistical contributions. However, tumor size and histologic type did not show any statistical significance. The reduced model was determined by Bayesian information criteria. Final model was selected considering both the statistical significance and Bayesian information criteria. The nomogram displayed in Figure 3 was built based on the final model. When using this nomogram, first locate the patient's variable value on each variable axis and draw lines upward to determine the point of each variable, then locate the sum of these points on the total points axis, finally draw a line downward to predict the 10‐ and 20‐year probability of death resulting from PTMC. In the 10‐fold cross‐validation, the discrimination (AUC) was 0.83 (95% CI, 0.79‐0.87) and 0.80 (95% CI, 0.76‐0.83) for 10‐ and 20‐year death, which indicated that the discrimination accuracy of the model is good. The AUC across all times is shown in Figure 4. The calibration plot of the 10‐year and 20‐year cumulative incidence function is displayed in Figure 5. The curve close to the 45‐degree line indicated acceptable agreement between predicted and actual probability of death resulting from PTMC.

**Table 3:** Proportional subdistribution hazard models of probabilities of death resulting from thyroid cancer

Characteristic	Full model		Reduced model		Final model	
	*β* coefficient	*P*	*β* coefficient	*P* a	*β* coefficient	*P*
Age	0.076	<.001	0.075	<.001	0.075	<.001
Male	0.303	.03	0.323	.02	0.306	.03
Race						
Black	0.502	.04	0.471	.06		
Other	0.319	.08	0.336	.07		
Tumor size	−0.019	.39				
Histologic subtype						
8260/3: Papillary adenocarcinoma, NOS	−0.268	.11				
8341/3: Papillary microcarcinoma	−0.436	.15				
8340/3: Papillary carcinoma, FV	−0.254	.15				
8342/3: Papillary carcinoma, oxyphilic cell	−9.715	<.001				
8343/3: Papillary carcinoma, encapsulated	−9.190	<.001				
8344/3: Papillary carcinoma, columnar cell	0.055	.94				
Extent of tumor						
Capsular extension	0.378	.25	0.394	.22	0.384	.23
Further extension	0.840	<.001	0.914	<.001	0.915	<.001
Lymph node involvement	1.318	<.001	1.435	<.001	1.423	<.001
Radiotherapy	0.320	.05				

Abbreviations: FV, follicular variant; NOS, not otherwise specified.

a A model selection technique based on the Bayesian information criteria was used.

## 4 DISCUSSION

In the present study, we found that 5‐year, 10‐year, and 20‐year probabilities of death resulting from PTMC, which is diagnosed as the first primary malignancy, were 0.3%, 0.6%, and 1.4%, respectively. The low mortalities indicated that PTMC could be considered a clinically very low‐risk cancer type. However, the 5‐year, 10‐year, and 20‐year probabilities of death resulting from other causes of these patients were 2.3%, 6.0%, and 10.6%, respectively, nearly 10‐fold higher risk than patients dying from PTMC. Given the excellent prognosis of PTMC, competing risks of death resulting from other causes are a crucial problem when evaluating the probability of death resulting from PTMC.

The occurrence of competing risk event prevents the occurrence of the event of interest. Thus, death resulting from other causes can be regarded as competing risk events in our study. Censoring those patients would result in informative censoring, lead to bias, and increase the estimates of incidence when researchers use the Kaplan‐Meier survival function. There are mainly two different regression models available to handle the competing risks: cause‐specific hazard model and proportional subdistribution hazard model. Patients who experience a competing event are removed from the risk set of the former model. In the latter model, such patients are kept in the risk set. The cause‐specific hazard model is better suited for characterizing factors related to the event of interest, and the latter model is often constructed to predict the probability of experiencing the event of interest.^16^ Given the primary objective of the present study, we used the Fine and Gray proportional subdistribution hazard model to construct models and the nomogram. Competing risk models for predicting some relatively indolent tumors have been published recently, such as prostate cancer,^17^ breast cancer,^18^ and also thyroid cancer.^19^ As far as we know, no studies have been reported on constructing a competing risks model to estimate the likelihood of death resulting from PTMC.

The treatment of patients with PTMC has been controversial between surgical treatment and active surveillance in recent years.^20^ Quantified estimates of the prognosis for a specific patient based on clinicopathological information are useful to weigh treatment benefits and risks. Several scoring systems have been proposed for thyroid cancer, such as AGES, MACIS, and AEMS scoring systems.^21^, ^22^, ^23^ Unlike these scoring methods, the nomogram is a graphic tool based on the statistical model which provides a numerical prediction of a clinical outcome for the individual patient and has been considered better than the conventional staging and scoring systems in some tumors.^24^, ^25^, ^26^ The nomogram built in our study provides clinicians with a practical tool for predicting the probability of death resulting from PTMC because clinicopathological variables involved in our final model are easily accessible to any clinician. In addition, our nomograms displayed favorable discrimination and calibration. It is worth noting that PTMC is indolent, and a 5‐year period is too short to detect death from the cancer. We believed that the prediction of death resulting from PTMC at 10 and 20 years would be more ideal.

Our study identified some independent factors that could predict the likelihood of death resulting from PTMC, such as age, sex, extent of tumor, and lymph node involvement. Age has been widely accepted as a prognostic determinant of death resulting from differentiated thyroid carcinoma. Some thyroid carcinoma staging systems treated age as a dichotomous variable. However, recent studies with large sample size indicated that patient age was a significant factor related to death resulting from papillary thyroid carcinoma in a linear fashion, without specific cutoff stratifying survival difference.^27^ It is more proper to consider age as a continuous variable when constructing nomogram.^28^ The morbidity of thyroid carcinoma is higher in women, but the cumulative incidence of death resulting from PTMC was two‐ to threefolds in man. Considering the importance of the prognostic factor, the capsular extension was incorporated in the nomogram though no statistical significance was found. Moreover, tumor size did not have significant impact on the cumulative incidence of death resulting from PTMC both in the Gray's test as a categorical variable or in the proportional subdistribution hazard model as a continuous variable. Nguyen et al also reported a consistent finding that there was very little change in thyroid mortality until the tumor size exceeded 2.5 centimeters.^29^ In addition, race, histologic type, and radiotherapy were also removed from the model.

The probability of death resulting from PTMC is low. Studies conducted in a single institution are usually powerless to determine significant prognostic factors for PTMC due to the uncommon death events and limited follow‐up periods. Our study extracted patients with PTMC from the SEER program which was a population‐based cancer database. The population‐based design, adequate sample size, and long‐term follow‐up enhanced the strength of our study. Therefore, estimating the prognostic factors of death resulting from PTMC is feasible in the present study. Moreover, the reliability and applicability of the outcomes from a population‐based cohort study are more superior.

Despite the above strengths, we acknowledge some limitations in our study. SEER database has a few inherent limitations, such as coding errors, limitation on the treatment, and lack of information about disease recurrence. Furthermore, we excluded the patients who did not undergo operation in the present study. However, a study based on patients with thyroid carcinoma from the SEER program showed a lower overall survival rate for the nonsurgical group due to advanced age and stage.^30^ Predictions of operable patients from the model which are built on the entire surgical and nonsurgical groups might be considered biased. The calibration plot of 20‐year thyroid cancer death from the validation was less than ideal, which is also a limitation of our study.

## 5 CONCLUSION

The present study provides useful information about the cumulative incidence of death resulting from PTMC based on a large, population‐based cohort. Patients with PTMC have excellent survival. Our nomogram constructed based on the competing risks model performs well and can be treated as a practical tool for clinical prognosis prediction.
